# Biomechanics of Human Motion and Its Clinical Applications: Instrumented Gait Analysis

**DOI:** 10.3390/bioengineering12101076

**Published:** 2025-10-03

**Authors:** Gordon Alderink, Sylvia Õunpuu

**Affiliations:** 1 Department of Physical Therapy & Athletic Training, Grand Valley State University, Grand Rapids, MI 49503, USA; 2 Connecticut Children’s Medical Center, University of Connecticut School of Medicine, Hartford, CT 06032, USA; sounpuu@connecticutchildrens.org

**Keywords:** clinical instrumented gait analysis, three-dimensional, motion capture, inertial measurement units, kinematics, kinetics, biomechanical models, electromyography, pedobarography, machine learning

## Abstract

A review of the methods and applications of marker-based and markerless-based motion capture and inertial measurement units for clinical gait analysis is offered to provide readers with an important historical and legacy-guided perspective. Advantages and limitations of these methods are delineated in light of Cappozzo’s ‘considerations on clinical gait evaluation’ and Brand and Crowninshield’s ‘comment on criteria for patient evaluation tools’. Critical summaries of each manuscript that make up this Special Issue reflect consideration of the notable comments by the legacy biomechanists who had the insights to frame important issues.

## 1. Introduction

The focus of this Special Issue was to examine the application of biomechanics as it relates to clinical instrumented gait analysis (IGA). Interest in the mechanics of locomotion, in general, but human gait in particular, has been notable for hundreds of years. In more modern times, it is clear that even minor impairments in gait can affect the quality of life, which has led to the development of specialized tools to measure and analyze normal and pathological gait. The modern gold standard used to take measures of gait include video motion capture (MOCAP) with instrumented measures of three-dimensional kinematics of the lower and upper extremities, pelvis and trunk, ground reaction and net joint kinetics, electromyography (EMG) of key muscles of the lower limbs, and plantar pressure. Although observational and quantitative measures of gait have been performed in research settings, beginning with the work of Borelli in the 17th century, clinical gait analysis only became feasibly useful in the past 50 years with advances in instrument resolution and computer power. For this Special Issue, we called for papers that would highlight the clinical applications of instrumented three-dimensional analysis of normal and pathological gait. Our call also included the desire for papers on advancements in biomechanical modeling and the application of MOCAP, EMG, biplane fluoroscopy, and inertial measurement units that had clinical applications. The purpose of this editorial will be to (1) review the evolution of the science and art of clinical gait analysis and (2) introduce the research included in this Special Issue.

## 2. Historical Overview of Clinical Gait Analysis

Early systematic studies of human locomotion began with Borelli’s work in the 17th century [1]. Based on Newtonian principles of mechanics, Borelli defined distinct phases of the gait cycle, and the primary muscles that controlled the lower extremities and the body’s center of mass during locomotion [1]. Later Wilhelm and Eduard Weber were able to better quantify measures of the stance and swing phases of locomotion in the 19th century [1]. In their broad historical review of gait analysis, Al-Zahrani and Bakheit [1] described the contributions of important pioneers, e.g., Marey, Muybridge, and Braune and Fischer, in the late 19th and early 20th centuries, who made two-dimensional (2D) and three-dimensional (3D) gait kinematic measures using photographic methods and light-emitting markers. The pioneering research of these men provided the foundation for Inman and his colleagues in the 1940s and 1950s to develop the synchronization of kinematic, kinetic (i.e., force plates), and electromyographic (EMG) data in the testing of normal and pathological, e.g., amputee, post-stroke, etc., gait [1]. Later refinements in motion capture equipment included the development and availability of the Vanguard Motion analyzer and SELSPOT systems in 1972 and 1976, respectively [1]. Although more advanced, the Vanguard and SELSPT were limited by the need to manually digitize anatomical markers and the donning of cumbersome power packs, respectively [1]. Today’s high-speed opto-electronic motion capture systems (MOCAP), such as VICON, CODA, Qualisys, etc., automate the location of retro-reflective or active anatomically placed markers and synchronize 3D kinematic measures with kinetic, multi-channel surface and fine-wire EMG, 2D video, and planar pressure measures of gait. Because MOCAP systems have dominated clinical gait analysis since the late 1970s and early 1980s, the data they produce are presently considered ground truth, i.e., the gold standard. Opto-electric motion capture systems allow gait analysis to shift from primarily research to clinical applications due to the reduced time to process data and inconvenience for patients during measurement.

Stebbins et al. [2] recently updated the status of clinical gait analysis. They noted that clinical gait analysis has taken “great leaps forward” since 1973. One piece of evidence of this was comparing clinical gait analysis citations over a nearly 50-year time period. Accessing Google Scholar for the year 1973, using the key phrase “gait analysis”, revealed a pittance of 28 results (with only 8 being relevant to human gait analysis), whereas a similar search for the year 2020 identified 10,200 results. Paralleling the increase in scholarly citations of gait analysis, the number of research and clinical gait laboratories worldwide has become widespread: (1) 50 in Australia/New Zealand, (2) 15 in the United Kingdom and Ireland, (3) 86 in North America, (4) 28 as part of the Francophone society, (5) 34 as part of the Italia society, and (6) 24 in the German speaking society. In their review, Stebbins et al. provide a general description of a typical gait analysis laboratory, including lab set-up and equipment used, patient preparation, procedures for the post-processing of measurement data, use of biomechanical, e.g., conventional (or Plug-in Gait), and forward dynamic/musculoskeletal models, and data interpretation. They noted several advances and advantages of opto-electronic MOCAP systems including the introduction of wireless EMG systems, the ability to track a multitude of anatomical markers spaced within 1 cm of each other with incredible accuracy, and significant improvements in data capture, real time data imaging, processing time, and graphical display capability. They also noted improvements in data interpretation related to better inter-observer reliability and removal of some regional variation in treatment recommendations. Despite these changes, general access to clinical gait laboratories remains relatively limited, the potential benefits of routine incorporation of musculoskeletal modeling into clinical gait analyses has not been fully realized, the use of automated identification of gait deviations is not available, and laboratory-based clinical gait analyses may not represent the “real world”. Although Stebbins et al.’s review provided a few references on the technical developments associated with gait analysis, this Editorial will expand their review by providing more detail.

## 3. Methods and Validation; Applications in Clinical Gait Analysis

### 3.1. Marker-Based Clinical Gait Analysis

According to Cappozzo, locomotion is achieved by the coordinated movements of the body segments employing a complex interplay of external and internal forces, which is best described using classical mechanics. He suggested that the quantitative description of the mechanical aspects of walking was referred to as gait analysis [3]. Davis expanded this by defining clinical gait analysis as the (1) measurement of fundamental biodynamic parameters, (2) organization of the basic measures into an information set, (3) interpretation of the compiled data set with respect to identification of deviations from normal patterns or values, (4) speculation about cause/effect, i.e., of abnormalities, and (5) recommendations for patient treatment options [4]. Davis noted that the approach used in their clinical gait laboratory was used in the assessment of pathological gait where motions were complex, multi-planar and distorted relative to a fixed observer, i.e., observational gait analysis, for children with cerebral palsy, amputation, degenerative joint disease, to name a few. He also noted the usefulness of the ability to document gait-related changes following intervention, e.g., orthopaedic surgery, selective dorsal rhizotomy, etc. Many others have described the aforementioned well-established methods used to gather data, which include physical examination of the patient, marker placement, motion capture, establishment of laboratory, technical, and anatomical coordinate systems, and the use of force platforms, dynamic kinesiological surface and fine wire EMG, and plantar pressure measures [3,4,5,6,7,8,9,10,11,12,13,14,15]; we note the notable work on the application of fine-wire EMG in clinical gait analysis by Jacqueline Perry, as reviewed by Sutherland [6].

Despite the consensus on method, several have highlighted the limitations related to marker-based MOCAP. Cappozzo [3] believed that, although the gait evaluation should include a description of the symmetry and simplicity of movement, maintenance of balance, mechanical load on tissues, and energy expenditure as a first step, a synthesis of the multifaceted data was complex and needed to be made relevant for the speculation used in clinical decision making. He further suggested that biomechanics research must overcome the stage where it only supplies information about how one walks and begin to answer the relevant whys, i.e., based on the general theories that governed the data. Sutherland [6] provided an extensive review of the development of kinesiological EMG for clinical gait analysis but noted that despite the advantages of using fine wire, i.e., minimizing cross-talk, the use of surface EMG was more clinically amenable. Sutherland noted that the determination of joint kinetics, i.e., net internal moments and power, enhanced the value of evaluating joint kinematics [7,8]. On the other hand, accurate and reliable marker placement, better methods for defining joint centers, especially the hip joint center, and addressing soft tissue artefact (STA) needed to be addressed [7,9,14,15]. Chiari et al. [13] summarized the cost of instrumental errors, including issues related to camera calibration, marker image processing and recovery of missing marker data, and smoothing and filtering of marker position data related to opto-electric stereophotogrammetry. They described methods to improve the accuracy and precision of stereophotogrammetric systems, which included recommendations for the minimization of error propagation from using a cluster of external markers. Others [9,14,15] addressed issues related to ameliorating STA and the sensitivity of determining joint kinematics to the definition of joint axes and precision of anatomical landmark determination. Leardini et al. [14] noted that reduced kinematic accuracy and precision related to STA was greater than the instrumental error. They noted that only sagittal plane motion about the hip, knee, and ankle could be determined reliably and concluded that reliable estimation of 3D skeletal system kinematics using skin markers had not yet been achieved. They suggested that inclusion of joint constraints into a more general STA minimization approach may provide an acceptable conclusion. Della Croce et al. [15] concluded that the effects of anatomical landmark position uncertainty on the definition of anatomical frames can lead to kinematic error propagation in joint rotation calculations out of the sagittal plane, a problem that needed to be addressed if gait analysis could be useful clinically. In addition to errors associated with STA and anatomical landmark accuracy and consistency, kinematic errors have also been associated with partially or totally occluded markers [16] and errors in the dimensions of the model’s body segments (model scaling) [17].

Besides the variability issues in instrumented gait measures [10,18], several non-technical concerns have been identified [18,19,20]. Although Simon [10] noted that IGA had its greatest impact as a test for individuals with cerebral palsy and was also useful for medical conditions in rheumatology, orthopaedics, endocrinology, and neurology, several other concerns were highlighted. For example, clinical gait analysis (1) was not widely used where it was needed, i.e., neuromuscular disorders, (2) some clinicians questioned whether this test was needed for patient treatment planning, (3) research had shown that IGA data did not appear to influence treatment outcomes by, for example, decreasing follow up care, (4) it was considered experimental by 3rd party payers in the United States, (5) testing was time consuming, (6) clinical reporting was a concern; that is, clinical reports were not produced in a timely fashion, were long, and in a format that was confusing for some clinicians, and (7) the tests were costly. Suggestions to address these concerns included increasing testing efficiency, computer assisted gait data analysis and report generation that was more user-friendly, and analytical techniques for gait assessment, e.g., neural networks [10,19].

Despite its limitations, marker-based MOCAP has the capability of providing clinicians with accurate and reliable gait data [4,9,20,21], partly related to improvements in the biomechanical models used and better management of soft tissue artifact [9]. Improvements in all aspects of IGA emerged partly due to the actions of key individuals involved in clinical gait analysis but also as a result of requirements of accrediting agencies [22,23,24,25], which have been active for the past two decades in the United Kingdom, Asia-Pacific, Europe, and the United States. Clinical gait laboratories who are accredited and have developed continuous quality improvement policies and procedures will lead in the enhancement of technical and clinical standards. To date, improvements have been shown to be effective in clarifying medical diagnoses and enhancing treatment recommendations [26,27,28,29,30]. For example, Wren et al.’s [28,29] systematic reviews showed that the literature between 2011 and 2020 related to the clinical efficacy of 3D IGA had grown substantially, showing continued improvement of data collection and interpretation, as well as understanding of gait pathology and treatment. In addition, they identified a small subset of studies that clearly demonstrated the efficacy of IGA in changing and reinforcing treatment decisions, increasing clinicians’ confidence in treatment planning, increasing agreement among clinicians, and the potential to improve patient outcomes.

### 3.2. Markerless-Based Clinical Gait Analysis

Although research using this methodology did not make this Special Issue, it is important to review it because of its impactful entrance into the clinical world. As noted above, several non-technical limitations in marker-based MOCAP may have hampered its wholesale utilization in clinical settings. Although marker-based systems are cheaper to acquire and run compared to biplanar stereophotogrammetric or videoradiography, they are still generally very costly for many clinical applications, and highly trained personnel are required operate them [10]. Additional limitations include participant preparation time, potential for erroneous marker placement, less amenable in more challenging data collection environments, e.g., outdoors, sports competition, etc., and physical and/or psychological constraints that may influence normal movements. Because of these limitations, fully automated, non-invasive markerless motion capture approaches have been developed and are currently being used in some sport and rehabilitative environments. Although markerless-based motion capture bases its determination of joint kinematics and net joint moments/powers on the same rigid body assumptions as marker-based approaches, it has the advantage of collecting data with fewer set-up constraints and in non-laboratory situations without long participant preparation time. Thus, it appears that markerless motion capture may have provided a solution for a common dilemma faced by clinical biomechanists, i.e., the trade-off between accuracy (laboratory-based analyses) and external validity (field-based analyses). However, before markerless motion capture can be used clinically with confidence, the construct and concurrent validity of its output must be substantiated relative to ground truth methods. Although fluoroscopy, Roentgen stereophotogrammetric techniques, and use of intra-cortical bone pins have produced joint kinematic and kinetic data that are considered the gold standards [31,32], their limitations, i.e., pre-testing preparation, need for significant user expertise, are laboratory-based, predisposition to harmful side-effects, and invasiveness, preclude their clinical relevance as ground truth. Therefore, marker-based MOCAP remains the default gold standard. Before we review the research that has examined the validity and reliability of markerless motion capture in gait analysis, let us briefly review how it works.

Markerless systems have some similarity to marker-based motion capture, i.e., use of cameras, need for optimal lighting configuration, and conferring the configuration of a skeletal model, but are challenged by the difficult process of gathering information from images rather than anatomically placed markers or marker clusters. The four major components of a markerless capture system are the (1) cameras, (2) representation of the human body, i.e., body model, (3) image features, and (4) algorithms used to determine the parameters (shape, pose, location) of the body model. In general, markerless motion capture uses standard video and relies on deep learning-based software (pose estimation algorithms) to describe human posture/movements for each individual image within the video, or videos from multiple cameras [31,32].

Two main types of camera hardware are used: depth cameras or standard video cameras, which can be used in single (monocular) or multiple-camera set-ups. Depth cameras record standard video but also record the distance between each pixel and the camera (depth). Depth cameras are relatively cheap and accessible but may not be as valid as marker-based systems and have limitations on capture rate, capture volume, and data collection may require controlled lighting conditions. Standard video hardware may have similar capture volume limitations as marker-based systems and high-speed video cameras require much brighter lighting. However, markerless motion capture is not limited by sunlight or multiple systems running simultaneously and zoom lenses or high-resolution video enable data collection from long distances, and low-cost systems, e.g., webcams or smartphones, can facilitate motion capture by clinicians (and coaches) in real world applications [32].

After video data is collected, pose estimation algorithms are used to detect and extract joint center locations. Pose estimation typically uses machine learning techniques that allow them to recognize patterns associated with anatomical landmarks. The algorithms are trained using large-scale datasets that provide the anatomical points of interest. Training a pose estimation algorithm usually requires the creation of a dataset that contains thousands of manually labeled key points. Deep learning estimation algorithms perform mathematical calculations on each image in the training set using a layered network, i.e., convolution neural network, that consists of many layers where the output of one layer becomes the input of the next layer. As a result, the pose estimation algorithm learns to identify key points, e.g., joint centers, as patterns of pixel color, gradient, and texture from the training data. The distances between manually labeled and estimated key point locations are then examined by an optimization method. This is an iterative process using the entire training set until differences between each iteration becomes negligible. Finally, the post estimation algorithm is tested on new images and compared to manually labeled data or marker-based joint center locations to check how well it performs on images it has never seen; thus, deep learning-based estimation will only be as good as the training data it used. OpenPose and DeepLabCut are pose estimation algorithms commonly used in biomechanical applications [31,32].

As opposed to marker-based systems that rely heavily on hardware to extract segment poses (location and orientation), markerless motion capture uses software to process the complicated image data obtained by standard video hardware. Because of this, pose estimation algorithms like OpenPose have been trained to extract only two points on each segment, i.e., proximal and distal joint center locations, whereas three points (e.g., proximal and distal end of a segment, and a third point placed somewhere else on the segment) are required to calculate six degree-of-freedom (DoF) motion. To determine 6DoF joint motion, two key points can provide information about the sagittal and frontal planes, while the third key point is needed to determine rotation about the segment’s longitudinal axis. Therefore, markerless methods that only identify joint center locations are limited to 5DoF, which permits only two-dimensional (2D) planar joint angles. This limitation can be overcome by (1) combining 5DoF methods with musculoskeletal modelling to constrain the movement that allows the estimation in 6DoF or (2) manually labeling training data with an additional third key point location on each segment [32]. Both DeepLabCut and Theia3D [33] have solved the 5DoF limitation by using multiple synchronized cameras and deep learning algorithms to create precise 3D skeletal models and extract detailed kinematic data.

Markerless motion capture has been available for approximately 10 years and there have been concerted efforts to test the validity and reliability (both intra- and inter-method) of several different systems, e.g., OpenPose, etc., against the default gold standard, i.e., marker-based MOCAP. At this point, there is no clear consensus regarding the accuracy, validity, and reliability of the markerless systems used in gait analysis. Yet, a plethora of such research is available, including several recent systematic reviews. Our purpose in this Editorial will be to summarize the results of the most recent systematic review and several studies that examined the use of markerless motion capture in clinical settings. However, before we examine the details of these works, it is necessary to operationalize several important statistical concepts: accuracy, validity, and reliability. This is necessary for a proper understanding of what is typically reported in manuscripts related to both experimental work and systematic reviews/meta-analyses.

*Accuracy: (i) how close the values of a given system are to the standard against which it is measured, and (ii) in this context, it refers to absolute agreement and one would be looking for differences, e.g., error (°), root mean square error, etc., that have been measured.*

*Validity: (i) assume concurrent validity where two methods are compared simultaneously when measuring relationships between variables, (ii) correlations between the two methods are determined, with high or excellent correlations, e.g., Pearson’s r, r > 0.75, between the two systems measures confirming concurrent validity, and (iii) in this context, validity refers to relative agreement,*

*Inter-trial reliability: Test–retest reliability of how stable measurements are when conditions remain unchanged over time,*

*Inter-rater reliability: consistency of measurements made by different raters/systems,*

*Intra-session reliability: consistency of measurements made by the same rater/system in a series of measurements made under the same conditions*

[34]

According to COSMIN guidelines, the intra-class correlation coefficient (ICC) is a valid reliability measure [35]. ICC values for reliability and validity can be interpreted as poor (<0.50), moderate (0.50–0.75), good (0.75–0.90) and excellent (>0.90). Pearson’s correlation assesses precision (relative agreement) and will be interpreted similarly as poor (r < 0.40), modest (r = 0.40–0.74), and excellent (r > 0.75).

Four review papers were identified and reviewed: narrative [31], scoping [32], and two systematic/meta-analysis [34,35]. We will summarize the results presented by Scataglini et al.’s review of 22 papers [34] for several reasons, where they (1) followed PRISMA [36] and COSMIN [37] guidelines, (2) selected studies on gait analysis by only markerless camera-based 3D and 4D motion capture systems of the lower body (pelvis, hip, knee, and ankle) and marker-based 3D motion capture systems of the lower body performed overground or on a treadmill, and (3) included studies that overwhelmingly included only healthy adults (which is also one its limitations). Nine articles investigated the reliability of 3D markerless-based systems compared to the gold standard: (1) for kinematic parameters, the ICC values indicated moderate to excellent inter-rater reliability for the knee and good to excellent reliability for the hip, but poor inter-rater reliability for the ankle; (2) excellent ICC values were reported for spatio-temporal gait parameters; (3) markerless system inter-trial reliability for hip, knee and ankle were poor, moderate to good, and moderate, respectively; in general, ICC values for hip, knee, and ankle inter-trial reliability were greater; (4) spatio-temporal inter-trial reliability was comparable between the two motion capture systems; and (5) intra-session reliability for walking speed was comparable between the two systems.

Nineteen studies assessed the concurrent validity and accuracy, comparing marker-based and markerless-based systems. For measures taken from treadmill walking, modest to excellent Pearson correlation coefficients were reported for the hip and knee in the sagittal and coronal planes; however, poor to modest correlations were found in transverse plane joint angles. There were significant inconsistencies noted among different studies. Pearson’s correlations for ankle kinematics ranged from poor to excellent. Spatio-temporal parameters showed modest to excellent correlations related to validity and accuracy. For overground data collection, (1) the validity of hip kinematics ranged from poor to excellent, (2) moderate to excellent agreement for knee range of motion in the sagittal plane was reported, but (3) all studies reported poor correlations between the two motion capture systems, and (4) good to excellent agreement was found between marker-based and markerless-based systems for spatio-temporal gait parameters.

Summary: Marker-based and markerless-based systems are comparable in terms of accuracy, concurrent validity, and reliability for spatio-temporal gait parameters; specifically, the meta-analysis revealed that the inter-rater reliability and concurrent validity for walking speed, step time, and step length resulted in good to excellent ICC values. Whereas kinematic values for the hip and knee in the sagittal plane were considered valid and reliable, measures of the hip and knee in the frontal and transverse plane, and ankle kinematics, failed to meet gold standard, clinically acceptable values. Customization and standardization of methodological procedures are needed before markerless-based systems can be considered for clinical application where serious patient interventions are at stake. Researchers also suggested that different patient populations needed to be studied. Let us now briefly examine a few studies that attended to a variety of clinical conditions.

Several publications compared the validity and reliability of markerless motion capture to that of maker-based systems in the evaluation of cohorts other than normal healthy adults. Horsak et al. [38] examined the concurrent validity of smartphone-based markerless motion capture using OpenCap [39] and marker-based MOCAP of lower limb kinematics in healthy adults under four gait conditions: physiological, crouch, circumduction, and equinus. They reported overall mean root mean square errors (RMSE) of 5.8° ± 1.8° with peak errors at 11.3° ± 3.9° for all joint kinematic variables. Repeated measures ANOVA with post hoc tests indicated significant differences in RMSE and peak errors between the four gait patterns, with the simulated pathological gaits showing the greatest errors. In a companion study of the same gait conditions, Horsak’s group [40] compared inter-trial variability between simultaneously recorded markerless and marker-based data. Compared to marker-based data, there was increased inter-trial variability for the markerless system ranging from 6.6% to 22.0% across the different gait patterns. These results suggested that the kinematic errors and increased inter-trial variability associated with markerless based systems were above clinically desirable thresholds.

Ripic et al. [41] compared lower extremity kinematics during overground walking between a traditional marker-based approach and a commercial multi-view markerless system [42] involving young and older adults, and adults with Parkinson’s disease. In their analysis, they also added data from a convenient sample of 35 healthy adults that yielded a total of 114 trials and 228 gait cycles. Time normalized waveforms, including 3D joint center locations, segment angles, and joint angles, were compared between systems using RMSE, range of motion difference (∆ROM), Pearson correlation coefficients (r), and interclass correlation coefficients (ICCs). RMSEs for joint center positions were less than 28 mm in all joints with correlations indicating good to excellent agreement. RMSEs for segment and joint angles showed the greatest agreement in the sagittal plane, with much larger errors in the transverse plane. ∆ROM differences were within reference values that typically characterize clinical groups, e.g., Parkinson’s disease, stroke, etc. The authors concluded that, although markerless solutions seemed promising for research and clinical settings, improvements were needed in pelvis tracking, markerless key point model definitions, and standardization of comparison study protocols.

Two studies compared data from marker-based and markerless-based motion capture systems that evaluated children with a range of diagnoses [43,44]. Wren et al. [43] evaluated data from 36 patients captured concurrently by a traditional marker-based motion capture system (Vicon Nexus) and a commercial markerless system (Theia3D). Multiple gait cycles were averaged from each patient and differences in kinematics (Theia-Vicon) were calculated over the gait cycle and evaluated using root mean square distance (RMSD), mean difference, and RMSD after subtracting the mean value across the gait cycle (RMSDoffset). Joint angles demonstrated similar patterns between the two systems and RMDS was <6° except for pelvic tilt, hip flexion, angle inversion, foot progression angle, and transverse plane rotation of the hip, knee, and ankle. However, RMSDoffset values were <6°. RMSD was greater for patients with foot deformities, those wearing orthoses or used assistive devices; however, RMSDoffset was <8°. In some cases, markerless system data exhibited greater trial-to-trial variability. Wishaupt et al. [44] compared the gait kinematics generated from marker- and markerless-based MOCAP systems from typically developing (TD) children and children with spastic cerebral palsy (CP). Statistical parametric mapping and paired *t*-tests were used to compare 3D trunk, pelvis, hip, knee, and ankle joint angles for both TD and CP, as well as the deviation from the normal in the CP group. Individual differences were quantified using mean absolute differences. Markerless kinematics failed to adequately track deviations in pelvic tilt and transvers hip rotation in children with CP.

### 3.3. Inertial Measurement Unit (IMUs)

The emergence of wearable technologies decades ago, including inertial measurement units (IMUs) that combine accelerometers, gyroscopes, and magnetometers, preceded the advent and exploration of the clinical application of markerless motion capture systems. Similar to markerless systems, IMUs are less costly, more portable, require less set up time, can provide more realistic evaluation of human movement, e.g., facilitate assessments outside traditional lab settings, and enable motion analysis in any environment [45]. Advances in miniaturization and accessibility have made such sensors a viable alternative to marker-based MOCAP. For example, inertial sensors are generally embedded with Bluetooth/wireless transmitters for real time data streaming using on-board SD cards for long term data recording. Finally, IMUs can be fixed on a single body segment, or a network of two or more inertial sensors can be used to retrieve data from multiple body segments simultaneously so that joint kinematics can be estimated [46]. Although IMUs may have fostered a shift in movement biomechanics and clinical gait analysis, two significant sets of limitations need to be acknowledged and addressed: (1) IMUs are limited by sensor drift which causes error accumulations and progressively reduces tracking accuracy over time; they are highly sensitive to sensor placement, considering factors such as soft tissue artifact, muscle movement, and attachment to non-bony areas that contributes to significant noise and comprises data accuracy and precision; they are influenced by external magnetic or metallic interference; and IMUs cannot directly measure kinetic variables, e.g., internal net joint moments and powers and (2) there is a lack of systematic research and reporting validating IMU output and clinical effectiveness [45].

Several systematic reviews have examined and reported on issues related to the methodology of IMU application in gait analysis, and reported on their validity and reliability [45,47,48,49,50]. Before we summarize the most important results of these papers, we will to recount the methodological approaches used for estimating lower extremity kinematics using IMUs. Joint kinematics has been defined as the relative orientation of two adjacent body segments; therefore, its estimate first requires the knowledge of the orientation of each body segment by using inertial sensors. In the 3D case, just as in stereophotogrammetry, the 3D orientation of the two adjacent body segments is needed. This is not straightforward because of a time-increasing “drift” of the angular displacement that affects the reliability of the sensor’s orientation. Fortunately, this can be controlled by the practice of fusing gyroscopic (angular velocity) and accelerometer signals, i.e., sensor fusion, where accelerometers correct the orientation computed from the numerical integration of the angular velocity with respect to gravity (roll and pitch angles). The lack of reference of the sensor’s orientation about the vertical direction (heading or yaw angle) is provided by a compass, which senses the local magnetic north, or magnetometer [46].

As noted, when estimating joint kinematics using IMUs, the sensor-to-segment axis alignment may be the most critical factor to consider since determining functional joint kinematics can only be accomplished by knowing the relative orientation between the anatomical axes of two adjacent body segments. Thus, assuming a direct approach, estimating functionally meaningful 3D joint kinematics using IMUs the orientation of the axes of the anatomical reference system representing the orientation of the body segment has to be known with respect to the orientation of the sensor-embedded reference systems; whereas an inverse approach enables full kinematic recovery without a sensor on each segment. This relationship is assumed to be time-invariant and is determined by a set of calibration procedures prior to the collection of walking data. The 3D joint kinematics are determined as the relative orientation between the proximal and distal sensor-embedded frames (expressed with respect to the same absolute reference frame). Specifically, one segment is expressed relative to the adjacent segment by multiplying the transposed rotation matrix of the proximal segment by that of the distal segment, resulting in a joint orientation matrix. Joint kinematics is then determined by decomposing the joint orientation matrix into three consecutive rotations about the specific anatomical axes following a specified order [46].

The 3D orientation of the sensor-embedded frame, with respect to an absolute reference frame, is typically provided by the manufacturer. However, securing the 3D kinematics from the sensor-to-segment alignment must be determined by the user via calibration procedures, choosing one of several approaches [51,52,53,54,55,56,57]. Two commonly used approaches, anatomical [52] and functional [51,53,54], have been described in some detail, yet two recent reviews examined the application of four common approaches [56,57]. Pacher et al. [56] included 54 studies in their systematic review, identifying manual, static, functional, and anatomical methods. With manual calibration, the examiner aligns one axis of the inertial-sensor coordinate system with the axis of the segment coordinate system. With static calibration, the participant adopts a particular posture, and the assumption is made that one of the segment axes is aligned with gravity measured by the accelerometer during this posture. For functional calibration, the participant of the experiment is instructed to perform pure rotations around the segment axes being identified. Finally, with anatomical calibration (also called the geometric approach), and similar to what is used in MOCAP, the examiner uses a device to locate anatomical landmarks or a segment axis with respect to a technical coordinate system associated with an inertial sensor placed on the device. The authors noted that often the different approaches are combined to define the relative orientation of the body segment with respect to the sensor-embedded reference system. Pacher noted that each method had advantages and disadvantages but the diversity of evaluating methodological robustness, i.e., validity and reliability, made it impossible to make a conclusion about the best method.

Vitali et al.’s [57] review of 112 manuscripts revealed four common approaches: (1) assumed alignment, i.e., anatomical, (2) functional, (3) model based, and (4) augmented. With the model-based method, anatomical axes are estimated by using either a kinematic model or statistical model of the joint. They noted that there was variation (1) in the estimates of the anatomical frames of reference between these four methods and also between the implementation within each category and (2) between the anatomical frames estimated from inertial motion capture versus those estimated by MOCAP. They suggested that the model-based methods may be most promising in that they did not report on the examiner’s ability to orient IMUs to the body segments or for participants to properly execute functional alignment movements as in assumed alignment and functional approaches, respectively. Vitali et al. also found it impossible to compare results across studies. Notably, the uncertainty about best practices in segment-to-sensor alignment suggests that much more conclusive research is needed in this area before IMUs can confidently be used for clinical gait analysis.

Although many studies have been published related to the examination of the validity and reliability of IMUs in clinical gait analysis over the past three decades, we will summarize the findings of three of the most recent systematic reviews to highlight what we know about these important topics [45,48,49]. Weygers et al.’s [49] wide-ranging review sought to evaluate the methodological requirements for IMU-based joint kinematic estimation to be applicable in clinical settings. Their findings led to several suggestions related to (1) future research related to the assumptions and prior information that are typically used to compensate for sensor limitations, (2) the need for adherence to general reporting standards suggested by the International Society of Biomechanics (ISB), (3) the continued development of biomechanical joint modeling, (4) considerations of measurement duration and environment, and (5) more research related to patient populations and impaired activities of daily living, e.g., sit-to-stand, stair-climbing, etc.

The systematic reviews by Kobsar et al. [48] and Prisco et al. [45] focused on the validity and reliability of spatio-temporal and 3D kinematic variables associated with gait analysis. One of the limitations of both reviews was that most of the studies predominantly included only healthy adults. Kobsar et al. included 82 papers in their systematic review and meta-analysis, with greater than 50% of them published between 2015 and 2020. For a validity study to be included, it must have assessed the concurrent validity of the IMU measured biomechanical gait outcomes as compared to the gold standard, i.e., marker-based MOCAP. Similarly, the reliability studies must have assessed between-day, within-day, or inter-tester reliability involving the same measure/device/placement with removal of IMU-measured gait outcomes. The biomechanical gait variables examined in this review included spatio-temporal gait parameters, segment or joint kinematics/kinetics, and other biomechanical outcomes, e.g., accelerations, stability, regularity, etc. Sufficient study quality and statistical outcomes needed for data pooling were available only for spatio-temporal gait parameters. Step time and stride time presented the strongest body of evidence for excellent validity and reliability. Good to excellent validity and reliability was noted for step and stride length, and stance and swing time. Contrary to mean outcomes, the validity and reliability of spatio-temporal variability and symmetry outcomes for step time, step length, and stance and swing time were poor to moderate. For kinematic measures in the sagittal plane, i.e., joint angles, consistent levels of good to excellent validity and reliability were reported. On the other hand, frontal and transverse plane joint angles demonstrated reduced overall validity and reliability and were generally found to be moderate to excellent. One of the most important findings of this review was the lack of high-quality evidence and appropriate statistical outcomes used in much of the research in this field. For example, many studies simply reported mean differences as a measure of validity and reliability, which only addresses the bias of the system. The authors claimed that information on best practices is limited and concluded that, although their findings demonstrated excellent validity and reliability of IMUs for the measurement of select spatio-temporal parameters, e.g., mean step/stride time and length during walking, caution is warranted for the use of IMU-measured joint angles out of the sagittal plane. Prisco’s work [45] included 32 papers published between 2014 and 2023. In addition to reporting on data from the examination of healthy adults, several of the studies reviewed included individuals with gait pathology, e.g., post stroke and Parkinson’s disease. Of those 32 papers, 11 focused on spatio-temporal gait parameters and 12 focused on joint kinematics. For validation metrics, 24 studies used correlation coefficients as the primary measures and only 7 used a combination of error metrics, correlation coefficients, and Bland–Altman analyses. Their findings showed that spatio-temporal parameters, such as cadence, stride time, stride cycle time, demonstrated stronger agreement compared to time spent in stance, double support, and swing. Kinematic parameters generally demonstrated consistently reliable levels of agreement, independent of sensor placement, the number of sensors used, or the validation metrics applied. Prisco’s review was limited by the heterogeneity of the included studies, particularly in sensor configurations, data processing methods, and validation metrics. They concluded that the use of IMUs offers a promising role in clinical gait analysis, tempered by the need for IMU technology and method to evolve in the development of standardization in positioning, refining signal processing algorithms, and improved validation protocols.

We conclude this section by referring the reader to a recent paper by Cereatti et al. [58] that provides the International Society of Biomechanics’ recommendations on the definition, estimation, and reporting of joint kinematics in human motion analysis applications using IMUs. They noted that the lack of standard IMU reporting guidelines compromises the interpretation and reproducibility of results, which has hindered the value of IMUs in the development of their clinical application. Cereatti et al. incorporated information from the biomechanics community, organized into five categories: sensor characteristics and calibration, experimental protocols, definitions of a kinematic model and subject-specific calibration, analysis of joint kinematics, and quality assessment, i.e., accuracy, concurrent validity, reliability, and context-specific validation. The authors suggest that their proposed recommendations are a first step for establishing best practice for the use of IMUs for human movement analysis.

## 4. Additional Comments on Clinical Gait Analysis

In 1981, Brand and Crowninshield [59] reflected on the necessary and sufficient criteria required for patient evaluation tools. Some may claim that clinical gait analysis is a tool used for the diagnosis of gait impairment, but Brand and Crowninshield suggested otherwise when they differentiated the terms ‘diagnose’ and ‘evaluate’. They claimed that to diagnose means to distinguish between diseases, which requires a history, physical examination, and other adjunctive tests, e.g., radiographs, blood tests, etc. This process is important in order to achieve the following:

1. Determine the severity of disease or injury, i.e., the assessment or evaluation.
2. To select from treatment options.
3. To monitor progress following intervention or in its absence.
4. Predict prognosis [18,59,60].

Evaluate means to place a value on something. Many medical tests achieve this as well, but instead of distinguishing diseases, these help to determine the severity of the disease, evaluate one parameter of the disease, or clarify the given diagnosis in some other way. Biomechanical tests, including quantitative clinical gait analysis, seem to be of this variety. On the other hand, clinical gait analysis can achieve more than create an understanding of the severity of a disease. For example, when gait pathology is defined by objective data, i.e., joint kinematics and kinetics, gait patterns emerge that are relevant to a specific medical diagnosis. Therefore, when the clinician has that knowledge, gait analysis may be able to contribute to a diagnosis, e.g., distinguishing between Charco-Marie Tooth versus idiopathic cavovarus or idiopathic toe walking.

However, in spite of the availability of advanced techniques to evaluate normal and pathological gait, Brand and Crowninshield noted that its use had not become widespread outside research labs, and believed that biomechanical evaluations of gait may never gain widespread clinical acceptance if they did not meet certain requirements for usefulness [18,59]:

1. The measured parameters must correlate well with the patient’s functional capacity.
2. The measured parameters must not be directly observable and semiquantifiable by the physician or therapist.
3. The measured parameters must clearly distinguish between normal and abnormal.
4. The measurement technique must not significantly alter the performance of the evaluated activity.
5. The measurement must be accurate and reproducible.
6. The results must be communicated in a form that is readily identifiable in a physical or physiological analog.

To Brand and Crowninshield, it was clear that most methods of gait analysis in 1981 did not meet all of their criteria, which may have explained its underutilization. In 2006, Baker [18] provided evidence and expanded on [59,60] and corroborated their claims, but then provided several recommendations that he thought would advance clinical gait analysis. Perhaps the two most important suggestions for making the interpretive component of a gait evaluation more objective were (1) the development of a general theory of how people walk and (2) clinical research to ascertain the output of particular interventions on groups of patients characterized by certain measurements. Baker’s first suggestion was motivated by comments expressed by Cappozzo [61], which followed his participation in a 1981 workshop on the clinical application of gait analysis. Cappozzo, as well as other conference participants, acknowledged advancements in the hardware and software used in gait analysis, but disappointment in ‘the state of the art’. According to Cappozzo, what was missing was a conceptual background of the method, that is, clinical gait analysis and evaluation was not supported by general theories. He concluded that (1) more efforts should be “devoted to speculation…try to interpret the phenomenon we have observed, we should try to identify, through generalization of single observations, the laws that govern them’, and (2) clinical gait evaluations “must overcome the stage where it supplies information about how man walks and begin to answer the relevant whys”. Beiser suggests that if we were to better integrate present gait analysis methods with general theories, clinical decisions may be based on the laws of physics and physiology rather than clinical experience and gait metrics of common interest conveniently measured [62].

Previously, we summarized key manuscripts that reported on the progress that has been achieved in clinical gait analysis research related to its efficacy and value [26,27,28,29,30], which appears to have initially addressed one of Baker’s suggestions. Yet, additional similar research is needed. However, the realization and use of a general theory and the incorporation of gait metrics with neuro-musculoskeletal models has not made significant inroads into the practices of clinical gait analysis [62]. Here, we will highlight a few seminal papers that provide the essential details of modeling processes. Interested readers should consult these papers for more information. The reviews by Hatze [5] and Buchanan et al. [63] provide a theoretical framework for the construction of inverse and forward dynamic models that are needed to explain the complexities, i.e., non-linear and system inter-related behaviors, associated with human movement in general, and gait in particular. Whereas Buchanan et al. [63] and others [64,65,66] provided the advantages and limitations of inverse dynamics and forward dynamics, Hatze [5] went beyond providing details of the methodology and models used in the quantitative analysis and optimization of human movement by discussing the fundamental principles upon which a unifying theory of human movement could be built. He suggested that the visible motion, i.e., joint kinematics, was a peripheral phenomenon created by the interplay of external and internal forces and moments acting on the skeletal system. It follows that the active internal moments are generated by the muscle forces controlled by the central and peripheral nervous systems, while the neural control functions are the consequence of complex processes within the hierarchical control pattern organization. The result of these complicated processes is a controlling system that produces purposeful and well-coordinated movements, e.g., normal walking. Furthermore, the system is capable of “learning”, i.e., evolving, using a set of optimizing procedures that is iterative. Hatze claimed that the goal-directedness of motion-generating neural control was related to the teleological nature of biological processes, and that the next step in the development of human movement science must be a precise axiomatic mathematical formulation of the teleological behavior of the human neurocybernetic system [5,64,65]. Following additional work, Hatze [65] described in more detail the challenges of what he terms the myoskeletal inverse dynamics and myocybernetic control inverse problems with respect to their ill-posed nature. He suggested that neither have unique solutions and, in fact, the myocybernetic control inverse problem was insolvable likely related to the inability of the pars intermedia, i.e., cerebellum, to control individual motor unit stimulation rates and recruitment patterns, but only whole muscles, or muscle groups by means of a “common drive”. He suggested that neuro-musculoskeletal models would have to account for the special neural circuits at the spinal cord level to decompose the common drive signal into motor unit recruitment patterns and stimulation rates that are specific to given mode of actions and certain optimization principles. Haggie et al. [67] provided essential detail to the ‘what’ and ‘how’ of Hatze’s projections by providing an overview of neuromuscular modeling and on how the integration of computational models, i.e., a more deterministic approach, might be used. They suggested that this integration must utilize the motor cortex, spinal cord circuitry, α-motorneurons, and skeletal muscle as input into the investigation of their respective roles in generating voluntary muscle action. As a result, they highlighted the challenges and opportunities related to the integration of a corticomuscular model, such as defining neuron connectivities, modeling standardization, and the application of models to study evolving behaviors. They rightly noted that so far, the field of computational biomechanics has focused on motor output, while the field of computational neuroscience has focused on the central nervous system, brain regions, and properties of neural networks, but for clinical gait analysis to advance, integrating neuroscience models of neurons and brain activity with biomechanical models of muscles in the body was necessary. Fregly [68] acknowledged that neuro-musculoskeletal modeling must progress from research to the clinical laboratory because of its potential to enhance the treatment of movement, i.e., gait, impairments, caused by common conditions such as stroke, osteoarthritis, cerebral palsy, etc. He enumerated the five following challenges: (1) movement data alone do not provide the answer, (2) every patient is unique, (3) people change over time, (4) validation is work, and (5) prediction of post-treatment is difficult and made recommendations for overcoming them. Furthermore, Fregly described the clinical, technical, collaborative, and practical needs and proposed solutions for neuromuscular and computational modeling to be useful clinically. He suggested that with enhanced model fidelity, personalization, and utilization, its selective clinical use will, in the short term, generate some initial clinical “wins” that might propel it “across the threshold of clinical utility and open up a new paradigm for treatment design”, and concluded that “if computational modeling and simulation can do for the design of movement impairment treatments what even a fraction of what they have done for the design of airplanes and automobiles, their clinical impact will be transformative”.

To this point, we have presented a historical and current overview of the methodologies and procedures predominantly used in clinical gait analysis. Four decades ago, clinical gait laboratory staff, e.g., physicians, physical therapists, and bioengineers, identified an underwhelming utilization of their services. Brand and Crowninshield [18] enumerated several possible reasons for this, with perhaps the most important being the complexity of the time-series data, i.e., 3D joint kinematics and kinetics, coupled with the clinicians’ lack of understanding. Although marker-based instrumented clinical gait analysis has made strides addressing these early failures, and research has demonstrated its clinical efficacy in selected pathologies, e.g., cerebral palsy, more recent recommendations for what is also necessary, e.g., increased used of biomechanical and computational models, in order to increase the utilization and effectiveness of clinical gait analysis has made little progress. Let us conclude this section with an exhortation by Besier [62]: “our focus needs to shift towards developing models that are agnostic to the measurement technique and capable of representing the underlying anatomy, physiology, mechanics, and motor control of a patient”.

## 5. What the Reader Can Expect in This Special Issue

We acknowledge that our ‘summary’ of the primary methodologies and procedures related to clinical gait analysis was lengthy, and perhaps ‘overkill’. However, we believe that it was necessary to provide a framework to show the reader what has been achieved and what is needed going forward. Our hope is this perspective will be considered by the reader as they peruse and critically analyze what is presented in this Special Issue.

### 5.1. Marker-Based MOCAP

Twelve manuscripts comprise this Special Issue, three of which involved the use of marker-based motion capture and two of these involved participants with gait impairment related to clinical pathology. In a retrospective analysis of children with heterogeneous limb impairment due to cerebral palsy (CP), Hara et al. [69] sought to use pre- and post-operative instrumented gait analysis data to explore potential predictors of the changes in pelvic rotation in a large cohort who had or had not been treated with a unilateral or bilateral femoral derotation osteotomy. Their results suggested that predicting changes in pelvic rotation was possible using gait kinematic data, especially for children with hemiplegic CP. Kim [70] noted the well-known relationship between symptomatic knee osteoarthritis (OA) and increased peak knee adduction moments (KAM) and shear forces during gait. He used a kinematic and kinetic analysis of self-selected speed level-ground walking of 35 individuals with knee OA to ascertain if differences in the foot progression angle (FPA), i.e., internally or externally rotated, altered the magnitude and timing of KAM. His results suggested that rehabilitation training that included voluntary changes in the FPA, either internal or external depending on the individuals’ walking characteristics, might be clinically useful.

The application of kinesiological surface electromyography (sEMG) in clinical gait analysis related to the treatment of cerebral palsy [6,10] typically consisted of the assessment/evaluation of the activation and timing of key lower extremity muscle, e.g., rectus femoris, medial hamstrings, medial gastrocnemius, and tibialis anterior, during locomotion. More recent reviews have discussed the methodological advantages and limitations of that application [71,72,73,74,75], as well as the need for further development [76,77]. Although this Special Issue does not include a paper presenting a traditional use of kinesiological surface EMG, Marino et al. [78] demonstrated an alternative use of sEMG in 20 healthy male children in their investigation of the possible differences in the motor control of walking under three walking conditions: (1) barefoot self-selected speed, (2) fast speed, and (3) normal speed carrying a backpack (with 12.5% body weight). They determined hip, knee, and ankle kinematics, spatio-temporal parameters, activity of bilateral tibialis anterior, lateral and medial gastrocnemius, rectus femoris, semitendinosus, biceps femoris, adductor magnus, and gluteus maximus and used non-negative matrix factorization (NNMF) to identify muscle synergy patterns, a method previously described and validated in the study of walking and running gait, and postural control by several research groups [79,80,81,82,83,84,85]. Although similar motor patterns characterized the control of all three walking conditions, the number of extracted synergies ranged from 3 to 5 across each condition. Although NNMF is not considered a neuro-musculoskeletal modeling method, it is an advanced analysis of sEMG that can begin to answer a “why question”, i.e., the cooperation within the central nervous system’s structural hierarchical organization of neurological control.

### 5.2. Inertial Measurement Units

Three manuscripts involved the use of IMUs in studies (1) to test the concurrent validity, relative to a marker-based MOCAP system, in assessing pelvis and trunk range of motion during overground walking [86], (2) to examine tibial accelerations related to treadmill running, comparing healthy controls and a fully recovered anterior cruciate reconstructed (ACLR) cohort [87], and (3) to test the validity of an IMU-based knee kinematics system in vivo [88]. Using 13 heathy participants who walked at self-selected speeds on level ground, Ali et al. [86] used IMUs on the sternum, sacrum, bilateral dorsum of the feet, and bilateral wrists, with concurrent use of the Helen Hayes marker set and MOCAP, to determine the validity of trunk and pelvis range of motion (ROM). They showed that all IMU measures were under 5 degrees for all measures, except the sagittal and transverse plane where ROM was within 10 degrees. There was greater variability of ROM measures for the pelvis in the sagittal and transverse planes, but the reliability of IMU and MOCAP measures were excellent overall. Further research is needed where IMU concurrent validity is tested in patient populations and in real-world environments.

Although vertical tibial accelerations (vPTA) appear to be an established IMU biomechanical metric to analyze runners related to overground and treadmill running, Hill et al. [87] sought to further investigate the use of tibial accelerations comparing ACLR and control cohorts. IMUs were secured to the rear part of shoes and shin bones and participants ran at self-selected constant speeds on a treadmill and concrete track. Besides measures of vertical (VPTAs), medial–lateral (mlPTAs), and anterior–posterior (apPTAs), ground contact time (GCT), and accelerations at initial ground contact were measured. Their results corroborated previous work showing greater peak tibial accelerations related to running on the track, regardless of health status.

Based on a series of experiments in their lab, Weber et al. [88] acknowledged that IMUs offer a promising alternative for assessing three-dimensional knee kinematics, making gait analysis more accessible. Their initial validation studies involving simulated tibiofemoral motion during level walking and the use of a custom-designed Rauch–Tung–Striebel IMU signal smoother demonstrated very small root mean square errors (RMSEs) for knee flexion/extension, abduction/adduction, and external/internal rotation. A follow-up study that posed their IMU algorithm against a MOCAP system using cadaver specimens during a loaded-squat showed RMSEs of 4.2° ± 3.6°, 0.9° ± 0.4°, and 1.5° ± 0.7° for knee flexion/extension, abduction/adduction, and external/internal rotation, respectively. In this Special Issue, Weber et al. presented the results of their tests of concurrent validity of their IMU algorithm versus MOCAP using healthy adult participants walking at self-selected speeds on a treadmill. Markers were placed on a rigid knee harness and IMUs were placed on elastic hook-loop bands secured around the distal femur and mid-shaft tibia (skin-mounted), as well as on the rigid harness. Before employing REFRAME (a REference FRame Alignment Method (REFRAME) to account for the effects of differences in coordinate system orientations), the skin IMU-based knee joint angles displayed a mean RMSE up to 6.5°, while mean RMSEs for harness-based IMUs peaked at 5.1°. After employing REFRAME, peak RMSEs were 4.1° and 1.52°, respectively. Although they found negligible differences between harness-based IMU and MOCAP knee kinematics after using REFRAME, they concluded that the obvious differences between the skin-mounted IMUs and MOCAP indicated that the use of a harness led to fundamentally different joint motion being measured, likely due to the movement of the harness at heel strike. It seems relevant to note that Weber’s findings relate to Brand and Crowninshield’s concern that “the measurement technique does not significantly alter the performance of the evaluated activity”. Certainly, the validation findings by Weber et al. need further study. In concluding this section, it is notable that two of the three studies that appear in this Special Issue attempted to further validate the use of IMUs clinically, which is important. However, as noted previously, it is equally important to test the use of IMUs in the implementation of neuro-musculoskeletal models that might be relevant clinically.

### 5.3. Modeling

In his narrative review, Piazza [89] surveyed available methods that may augment traditional IGA techniques, i.e., motion capture to determine 3D joint angles and the use of ground reaction forces and moments in inverse dynamics to estimate net internal joint moments and powers, that only indirectly provide information about the role of individual muscles in normal and pathological gait. That is, although sEMG directly measures muscle activations of individual muscles, joint motion information integrated with estimates of joint moments and powers can only inform us about the collective actions of muscles. Piazza discussed the challenges faced by clinical biomechanists including the need to estimate muscle moment arms, understand what he described as muscle “gear ratio”, and the need to use neuro-musculoskeletal models in the estimation of muscle-tendon lengths. He concluded his review by defining a type of muscle model referred to as muscle-induced acceleration analysis (IAA) and examples of its application in research related to the study of normal and pathological gait. The potential of the IAA (and induced power) method is that it can help elucidate how muscles are coordinated to produce a variety of movements in a smooth, coordinated, and efficient manner, and provide insight into causal relationships between muscle action and output movement. It is possible that these insights may help to clarify the complexity and redundancy of the neuromuscular system, as well as its limits under pathological conditions [90]. The power of IAA lies in its potential to contribute to a general theory of human movement and principles of neuromuscular control that drive movements, as called for by Baker and others [18,64]. Yet, caution is advised since attention also must be directed to the effects of the musculoskeletal model that is chosen, i.e., complex versus simpler [89,91].

Based on the significant prevalence of knee osteoarthritis (KOA), Zhang et al. [92] identified a need to accurately quantify internal knee contact forces, i.e., tibiofemoral contact force (TFCF)-related activities of daily living (ADL), e.g., walking, sit-to-stand, stand-to-sit, stair ascent, and stair descent in a cohort of healthy adults. Using 3D MOCAP synchronized with floor embedded force plates and incorporation of a subject-specific musculoskeletal model (based on the generic model in AnyBody software), they assessed the prediction accuracy by comparing the TFCF curves between the model predictions and in vivo measurements, and predicted the TFCF on the total, medial, and lateral knee compartments. The reported that TFCFTotal was the greatest during stair ascent and sit-to-stand, followed by stair descent, stand-to-sit, and walking. Prediction accuracy showed good agreements of TFCF curves between the model predictions and in vivo measurements for all five activities (RMSE: 0.216~3.11 body weight, R^2^ (coefficient of determination): 0.928~0.992, and Sprague and Geers metrics of combined error (C_E_): 0.048~0.141). Although the cause of KOA is multifactorial and this study’s results cannot speak to cause/effect, the results may provide insights into the biomechanics of selected normal ADLs and provide guidance for preventing and treating KOA. Here we have another example of the use of a musculoskeletal model, i.e., technique, that can provide additional and useful information and may also add to a general theory of KOA and ultimately improve treatment outcomes.

### 5.4. Machine Learning

First, we provide some context. Machine learning (ML) is a form of artificial intelligence (AI) and a complex process that enables a system to learn from data rather than through explicit programming. ML uses a variety of algorithms that iteratively learn from data to improve, describe data, and predict outcomes. As the algorithms digest training data, it is possible to produce more precise models based on that data. The disciplines of statistics, data mining, and ML all have a role in understanding data, describing the characteristics of a data set, and finding relationships and patterns in that data to build a model. Deep learning (DL) is a specific method of ML that incorporates artificial neural networks (ANNs) in successive layers in order to learn from data in an iterative manner. DL is especially useful when trying to learn patterns from unstructured data, involves complex neural networks, and is designed to emulate how the human brain works so computers can be trained to deal with abstractions and problems that are poorly defined. Finally, different ML techniques are required to improve the accuracy of predictive models. Three different approaches are being used: (1) supervised learning begins with an established set of data, a certain understanding of how that data is classified, and is intended to find patterns in data that can be applied to an analytic process; (2) unsupervised learning is suited for problems that require a massive amount of data that is unlabeled and conducts an iterative process of analyzing data without human intervention; and (3) learning is a behavioral learning model that receives feedback from the analysis of the data; that is, it is not trained with a sample data set, but learns through trial and error [93].

Instrumented, marker-based, gait analysis can be a source of big data that could be useful for providing a deeper understanding of the neuro-musculoskeletal impairment data it creates. These data sets include 3D joint motions of the trunk, pelvis, and lower extremities, net internal joint moments and powers, EMG activation patterns of several lower extremity muscles bilaterally, information on past medical and surgical history, radiographic interpretations, and physical examination measures. In the past two decades, ML has made significant inroads in the field of biomechanics and human movement science, e.g., clinical gait analysis. Therefore, the purpose of Dibbern et al.’s [94] scoping review was to demonstrate how ML and DL have been employed in the use and interpretation of instrumented, marker-based gait analysis data. The review only included papers that contained the following three components: (1) use of marker-based 3D gait analysis data, (2) analysis of data using ML techniques, and (3) the leveraging of ML to make predictions or classifications; 105 articles were included in the final review. The most common clinical conditions revealed in the review included cerebral palsy, post-stroke, and Parkinson’s disease; other conditions included healthy participants, those with KOA, runners, ligamentous knee injuries, patellofemoral pain, diabetes, and flatfeet. The most commonly used ML techniques included support vector machines (SVMs), cluster analysis after principal component analysis (PCA) data reduction, neural networks (NNs), and logistic regression. Other techniques identified included classical Bayesian, random forest, ensemble, fuzzy decision tree, multiple correspondence analysis, and statistical parametric mapping. The authors did not identify any studies that evaluated the clinical interpretation of gait analysis data from ML into their routine workflows. A problem identified in this review may be related to the lack of support networks for the integration and use of ML in a clinical setting. Thus, to gain confidence in the outcome of ML applications and their use clinically, use of explainable ML methods, e.g., explainable-AI (XAI) was recommended. Based on their review, the authors suggested that traditional ML techniques have promise for assisting in the analysis of 3D gait analysis data and its clinical application.

Motivated by a need to address the prevalence of injury-related falls in older adults, Marimom et al. [95] aimed to identify key features of normal walking using advanced statistical and ML methods. A gait analysis using one IMU secured onto the shoed forefoot of 32 healthy adults walking at two different speeds (2 km/h and 4 km/h) on a treadmill was conducted. Sixty-four statistical features and the application of eight ML classifiers were used to evaluate four gait phases: stance, toe-off, swing, and heel strike. Spider plot analysis revealed significant differences in the gait events that the authors concluded could be used to contrast individuals with ankle sprains or foot drop. They also demonstrated that the support vector machine (SVM) model, with an accuracy rate of 92.4%, could be used to differentiate gait events in healthy individuals. The authors suggested that their methods may have a role as a predictive model and be useful in the evaluation of gait-related disorders. Although the authors chose to use metrics that have been shown to correlate with a patient’s function, are not directly observable, and can distinguish between normal and abnormal, their method was not validated, nor could their results be communicated in a form that would be readily identifiable by a clinician [18,59].

### 5.5. Application of Plantar Pressure Measures to Characterize Antalgic Gait Associated with Hallux Valgus and Use of Gait Analysis to Study the Effects of Muscle Fatigue on Locomotion

Although related to clinical gait analysis, the final two papers to be reviewed do not fit into the previous categories. Chen et al. [96] tested 35 patients, randomly assigned to experimental (innovative full-contact insoles) and control (regular foot insoles) groups, with forefoot or plantar pain associated with hallux valgus. Participants were instructed to wear the orthotic six hours per day. Data were collected a baseline, and at 1 week, 2 months, and 3 months after the fitting. Foot pressures were measured with the Tekscan in-shoe pressure system, gait parameters were measured with the GAITRite walkway, and static balance was measured with force plates. After 12 weeks, those using the innovative orthotic demonstrated significantly: (1) reduced antero-posterior and area displacement based on static balance testing, (2) increased swing phase and a reduction in stance phase, and (3) reduced peak pressures at the 2nd metatarsal and medial heel, and increased contact areas in the midfoot. This research showed promising results which will need to be replicated and demonstrate how these tools could help clinicians better understand their treatment outcomes.

Wang et al. [97] were interested in what the literature has reported about how experimentally induced muscle fatigue would affect gait kinematics, kinetics, and muscle activation. The authors acknowledged the challenges in measuring the effects of fatigue since the influence of peripheral versus central factors needed to be considered, as well as whether body and muscle fatigue were induced in a laboratory or field setting. Consensus on the role of muscle fatigue on gait metrics has not been definitive because of the heterogeneity of fatigue protocols. Acceptable papers (n = 11) for this review included only those that used the following laboratory-induced fatigue protocols: isokinetic dynamometers, cycle ergometry, treadmills, and sit-to-stand tasks. Most studies reported an increase in step width, decrease in net internal knee moments, and reductions in muscle activation levels. Although the authors noted that reduced external validity, selection bias, and statistical power affected their study’s quality, knowing the compensation strategies used by individuals who are affected by muscle fatigue may help mitigate overall fall risk.

## 6. Conclusions

“We shall not cease from exploration, and the end of all our exploring will be to arrive where we started and know the place for the first time”: T. S. Eliot. This is not actually true about this Editorial, but mostly true. As we initiated and progressed with this project, it dawned on us that the scientists (theoretical, practical, and clinical) whose writings we reviewed were constantly in two worlds, the present and perceived future. That is, it seemed like they could actually see a “necessary and sufficient” future if clinical gait analysis were to reach its potential. We will conclude this Editorial with quotes from notables who, we think “set the pace” for the rest of us.

Brand and Crowninshield (1981) [59]: “In our opinion any evaluation tool will be useful (and thus gain widespread acceptance) if, and only if, the technique meets all of the following criteria.

1. The measured parameters must correlate well with the patient’s functional capacity.
2. The measured parameters must not be directly observable and semiquantifiable by the physician or therapist.
3. The measured parameters must clearly distinguish between normal and abnormal.
4. The measurement technique must not significantly alter the performance of the evaluated activity.
5. The measurement must be accurate and reproducible.
6. The results must be communicated in a form that is readily identifiable in a physical or physiological analog.

It is clear to us that most methods of assessing gait do not meet all of these criteria.”

Cappozzo (1983) [61]: “We biomechanicians have been working hard, during the last decades, designing new instruments, applying old analytical techniques to new problems…It must overcome the stage where it supplies information about how man walks and begin to answer the relevant whys… We should try to interpret the phenomenon we have observed, we should try to identify, through generalization of single observations, the laws that govern them.”

Cappozzo (1984) [3]: “It is notable that retrieving quantitative parameters related to gait is only the first step…a synthesis of the data is needed in order to supply clinically relevant information, and this step is complex and multifaceted and requires the speculative involvement of the clinician.”

Hatze (1984) [5]: “At the present stage of development of human movement science it has become clear that only an integrative systems approach combining appropriate models of the skeletal (executor), muscular (myoactuator), and neural (neurocybernetic) subsystems under the unifying principle of teleological systems behavior will lead to further progress. Such an approach permits the qualitative (topological) and quantitative description of motion characteristics such as rhythm, economy and efficiency of movement and will enable us to proceed towards a unified theory of human motion”.

Andriacchi and Alexander (2000) [9]: “A critical challenge for the future is to develop new and more powerful modeling and observation techniques”.

Simon (2004) [10]: “Current biotechnology research is seeking to address these problems by creating techniques to capture data rapidly, accurately, and efficiently, and to interpret such data by an assortment of modeling, statistical, wave interpretation, and artificial intelligence methodologies. The success of such efforts rests on both our technical abilities and communication between engineers and clinicians”.

Baker (2006) [18]: “There is still not an accepted general theory of why we walk the way we do…many explanations of walking address the mechanisms by which specific movements are achieved by particular muscles…a new methodology is developing to determine the functions of individual muscles (i.e., IAA). This needs further development and validation…Very recent work has started to show the potential of using models of the mechanisms by which people with pathology walk…The development of these models offers considerable promise for new clinical applications of gait analysis”, and finally, Besier (2025) [62]: “Our focus needs to shift towards developing models that are agnostic to the measurement techniques and capable of representing the underlying anatomy, physiology, mechanics, and motor control of a patient”.

This Special Issue’s contributions have demonstrated several clinical applications of gait analysis data and in many ways have responded to what our predecessors called for. However, much more work needs to be carried out, particularly in the integration of gait analysis metrics, e.g., joint kinematics, etc., and neuro-musculoskeletal modeling. We encourage the cohort of biomechanists and clinicians who will write the next chapter on clinical gait analysis to set their expectations high.
